# E-Bayesian Estimation of Chen Distribution Based on Type-I Censoring Scheme

**DOI:** 10.3390/e22060636

**Published:** 2020-06-08

**Authors:** Ali Algarni, Abdullah M. Almarashi, Hassan Okasha, Hon Keung Tony Ng

**Affiliations:** 1 Department of Statistics, Faculty of Science, King Abdulaziz University, Jeddah 21589, Saudia Arabia; ahalgarni@kau.edu.sa (A.A.); hokasha@kau.edu.sa (H.O.); 2 Department of Statistical Science, Southern Methodist University, Dallas, TX 75275-0332, USA; ngh@mail.smu.edu

**Keywords:** E-Bayes estimation, Bayes estimation, Chen distribution, type-I censoring scheme, balanced loss function

## Abstract

In this paper, E-Bayesian estimation of the scale parameter, reliability and hazard rate functions of Chen distribution are considered when a sample is obtained from a type-I censoring scheme. The E-Bayesian estimators are obtained based on the balanced squared error loss function and using the gamma distribution as a conjugate prior for the unknown scale parameter. Also, the E-Bayesian estimators are derived using three different distributions for the hyper-parameters. Some properties of E-Bayesian estimators based on balanced squared error loss function are discussed. A simulation study is performed to compare the efficiencies of different estimators in terms of minimum mean squared errors. Finally, a real data set is analyzed to illustrate the applicability of the proposed estimators.

## 1. Introduction

Many lifetime distributions have been proposed in the literature to analyze data with bathtub-shaped failure rates. Distributions with bathtub-shape hazard rate functions provide an appropriate conceptual model for some electronic and mechanical products as well as the lifetime of human beings. For a detailed review about bathtub-shaped distributions, one may refer to References [1,2,3,4,5]. In this paper, we exclusively focus on the two-parameter bathtub-shaped lifetime model introduced by Reference [6]. Reference [6] introduced a probability distribution that can give different shapes of hazard functions, including decreasing, increasing and bathtub shapes. The cumulative distribution function (cdf) of the Chen distribution is given by

(1) $$F\left(x;\theta ,\lambda \right)=1-e^{\theta (1-e^{x^{\lambda }})},\;x>0,\theta >0,\lambda >0,$$

and the corresponding probability density function (pdf) is

(2) $$f\left(x;\theta ,\lambda \right)=\theta \lambda x^{\lambda -1}e^{x^{\lambda }+\theta \left(1-e^{x^{\lambda }}\right)},\;x>0,$$

where θ>0 and λ>0 are the scale and shape parameters, respectively. The survival function, also known as the reliability function, is

(3) $$R\left(t;\theta ,\lambda \right)=e^{\theta (1-e^{t^{\lambda }})},\;t>0.$$

and the hazard rate function can be derived as

(4) $$h\left(t;\theta ,\lambda \right)=\theta \lambda t^{\lambda -1}e^{t^{\lambda }},\;t>0,$$

Recently, the Chen distribution has gained more attention among researchers because if its ability to model various shapes of the hazard rate. Reference [7] discussed the point and interval estimation based on progressive censoring. Reference [5] investigated the Bayesian estimation under progressive censoring using different loss functions. Reference [8] obtained Bayes estimators for progressively censored data based on a balanced squared error loss (BSEL) function. Reference [9] discussed Bayes estimation and prediction under progressive censoring. In this paper, we study the E-Bayesian estimation of the scale parameter, reliability and hazard rate functions of the Chen distribution based on type-I censored data. All of these estimators are obtained under the assumption that the shape parameter is known. It is worth mentioning here that this paper is the first attempt to estimate these functions of the Chen distribution using the E-Bayesian scenario. The balanced loss function (BLF) introduced by Reference [10] has the following form

(5) $$L_{\varphi ,\omega ,\rho_{0}}^{p}\left(\tau \left(\theta \right),\rho \right)=\omega p\left(\theta \right)\varphi \left(\rho_{0},\rho \right)+\left(1-\omega \right)p\left(\theta \right)\varphi \left(\tau \left(\theta \right),\rho \right),$$

where φ(τ(θ),ρ) is an arbitrary loss function when estimating τ(θ) by ρ and p(.) is a suitable positive weight function. Let $\rho_{0}$ be the parameter priori estimator of τ(θ) obtained from a frequentist method such as the least squares method and the maximum likelihood methods. Reference [10] discussed a general Bayesian connection between the case of ω=0 and ω>0, where 0≤ω<1. Let p(θ)=1 and $\varphi \left(\tau \left(\theta \right),\rho \right)={\left(\rho -\tau \left(\theta \right)\right)}^{2}$, using the results in Reference [10], the BLF reduced to the BSEL function as

(6) $$L_{\omega ,\rho_{0}}\left(\tau \left(\theta \right),\rho \right)=\omega {\left(\rho -\rho_{0}\right)}^{2}+\left(1-\omega \right){\left(\rho -\tau \left(\theta \right)\right)}^{2},$$

and the Bayes estimate of the function τ(θ) can be written as

(7) $$\rho_{\omega ,\tau ,\rho_{0}}\left(x\right)=\;\omega \rho_{0}+\left(1-\omega \right)E\left(\tau \left(\theta \right)|x\right).$$

To the best of our knowledge, all the studies in the literature investigated the estimation problems of the Chen distribution based on classical and Bayesian methods of estimation under different censoring schemes. Therefore, it is the first time studying the E-Bayesian estimation of the scale parameter and the reliability characteristics. We show later, based on simulation study and real data analysis, the significance of the proposed estimators over the classical and Bayes estimators. Furthermore, we provide a guideline for selecting the best estimation method for the scale parameter and the reliability characteristics of the Chen distribution. Our aim in this paper is to study the E-Bayesian estimation of the scale parameter, the reliability and hazard rate functions of the Chen distribution based on type-I censored data. We consider the gamma distribution as a prior distribution to the unknown scale parameter and obtain the E-Bayesian estimation based on three different distributions to the hyper-parameters. We compare the performance of different estimators via a simulation study as well as analyzing a real data set. The rest of the paper is organized as follows. In Section 2 we discuss the Bayesian estimation of the scale parameter, the reliability and hazard rate functions. The proposed E-Bayesian estimation is discussed in Section 3. Some properties of the E-Bayesian estimation method are studied in Section 4. The results of the simulation study are presented in Section 5. A real data set is analyzed in Section 6. Finally, the paper is concluded in Section 7.

## 2. Bayesian Estimation

In this section, suppose that *n* items are put on a life test simultaneously. The ordered lifetime of the *n* items are $X_{\left(1\right)}<X_{\left(2\right)}<\ldots <X_{\left(n\right)}$ and the life test is terminated at a fixed time τ. The number of observed failures before time τ is a random variable and these observations represent the type-I censored sample. By using of type-I censored sample from Chen distribution with cdf and pdf given by Equations (1) and (2), respectively, the likelihood function can be written as

(8) $$L\left(\theta ,\lambda |x˗\right)=\frac{n!}{(n-r)!}\;\theta^{r}v\left(\lambda ;x˗\right)e^{-T\theta },$$

where

(9) $$x˗=\left(x_{\left(1\right)},x_{\left(2\right)},\cdots ,x_{\left(r\right)}\right),\;v\left(\lambda ;x˗\right)=\lambda^{r}\prod_{i=1}^{r}x_{i}^{\lambda -1}e^{\sum_{i=1}^{r}x_{i}^{\lambda }},$$

and

(10) $$T\equiv T\left(\lambda ;x˗\right)=\sum_{i=1}^{r}e^{x_{i}^{\lambda }}+\left(n-r\right)e^{\tau^{\lambda }}-n.$$

In the current model, it is difficult to investigate the E-Bayesian estimation of shape parameter λ because of the complicated double exponents. When λ is known in the two-parameters lifetime distribution, the maximum likelihood estimator (MLE) of the parameter θ, can be derived in a closed-form as

(11) $${\hat{\theta }}^{ML}=\frac{r}{T}.$$

It is well known that the MLE of the parameter does not exist when r=0, therefore, the censoring time τ should set to be reasonably large to ensure r>0. The corresponding MLEs of the reliability function $R\left(t\right)$ and the hazard rate function $h\left(t\right)$ are obtained, respectively, from Equation (3) and Equation (4) by using (11), after replacing θ with its MLE, ${\hat{\theta }}^{ML}.$

For the Bayesian estimation of the parameter θ, the gamma conjugate prior density

(12) $$g\left(\theta |a,b\right)=\frac{b^{a}}{\Gamma (a)}\theta^{a-1}e^{-b\theta },\;\theta >0,$$

where a>0 and b>0, is used and the posterior density of θ based on the random sample, x˗, can be obtained from Equations (8) and (12) as

(13) $$q\left(\theta |x˗\right)=\kappa \theta^{r+a-1}e^{-(b+T)\theta },\;\theta >0,$$

where

(14) $$\kappa =\frac{{\left(b+T\right)}^{r+a}}{\Gamma (r+a)}.$$

Under the BSEL function, the Bayesian estimator of θ can be shown to be

(15) $${\hat{\theta }}^{BS}\left(a,b\right)=\omega \left(\frac{r}{T}\right)+\left(1-w\right)\left(\frac{r+a}{b+T}\right).$$

Under the BSEL function, the Bayes estimator of the reliability function can be obtained from Equations (3) and (12) as

(16) $$\begin{array}{ccc} {\hat{R}}^{BS}\left(t\right) & = & \omega e^{\frac{r}{T}\left(1-e^{t^{\lambda }}\right)}+\left(1-w\right){\left(\frac{b+T}{b+T+T^{*}}\right)}^{r+a}, \end{array}$$

where

(17) $$T^{*}\equiv T^{*}\left(\lambda ;t\right)=e^{t^{\lambda }}-1.$$

Similarly, under the BSEL function, the Bayes estimator of the hazard rate can be shown from Equations (4) and (12) as

(18) $$\begin{array}{ccc} {\hat{h}}^{BS}\left(t\right) & = & \lambda t^{\lambda -1}e^{t^{\lambda }}\left[\omega \left(\frac{r}{T}\right)+\left(1-w\right)\left(\frac{r+a}{b+T}\right)\right]. \end{array}$$

## 3. E-Bayesian Estimation Based on BSEL

According to Reference [11], the prior parameters *a* and *b* should be selected to guarantee that the prior g(θ|a,b) in Equation (12) is a decreasing function of θ. The derivative of g(θ|a,b) with respect to θ is

$$\frac{dg(\theta |a,b)}{d\theta }=\frac{b^{a}}{\Gamma (a)}\theta^{a-2}e^{-b\theta }\left[\left(a-1\right)-b\theta \right].$$

Thus, for 0<a<1, b>0, the prior g(θ|a,b) is a decreasing function of θ. Assuming that the hyper-parameters *a* and *b* are independent random variables and their density functions are $\pi_{1}\left(a\right)$ and $\pi_{2}\left(b\right)$, respectively. Then the joint bivariate density function of *a* and *b* can be represented,

$$\pi \left(a,b\right)=\pi_{1}\left(a\right)\pi_{2}\left(b\right).$$

When the BSEL function is used, the E-Bayesian estimate of θ (expectation of the Bayesian estimate of θ) is defined as

(19) $${\hat{\theta }}^{EBS}=E\left(\theta |\underline{X}\right)=\int_{\varrho }\int {\hat{\theta }}^{BS}\left(a,b\right)\pi \left(a,b\right)dadb,$$

where ϱ is the domain of *a* and *b* for which the prior density is decreasing in θ. ${\hat{\theta }}^{BS}\left(a,b\right)$ is the Bayes estimate of θ given by Equation (15). For more details, see Reference [12], Reference [13], Reference [14], Reference [15], Reference [16] and Reference [17].

### 3.1. E-Bayesian Estimate of the Parameter θ

Reference [18] used a uniform prior distribution for the hyper-parameter to study E-Bayesian estimation of the shape parameter of Pareto distribution. In this study, there are two hyper-parameters, *a* and *b*, and the properties of E-Bayesian estimates of θ rely on different distributions of the hyper-parameters *a* and *b*. In order to investigate the E-Bayesian estimation of θ, we consider using the following three joint distributions,

(20) $$\left.\begin{array}{cc} \; & \pi_{1}\left(a,b\right)=\frac{1}{sB(u,v)}a^{u-1}{\left(1-a\right)}^{v-1},\;\;\;0<a<1,\;0<b<s\;\\ \; & \pi_{2}\left(a,b\right)=\frac{2}{s^{2}B\left(u,v\right)}\left(s-b\right)a^{u-1}{\left(1-a\right)}^{v-1},\;0<a<1,\;0<b<s\;\\ \; & \pi_{3}\left(a,b\right)=\frac{2b}{s^{2}B\left(u,v\right)}a^{u-1}{\left(1-a\right)}^{v-1},\;\;\;0<a<1,\;0<b<s\; \end{array}\right\}$$

where u>0, v>0 and B(u,v) is the beta function. Therefore, E-Bayesian estimators of the parameter θ under BSEL function can be derived by using Equations (15), (19) and (20). The E-Bayesian estimator of θ under the BSEL function and based on $\pi_{1}\left(a,b\right)$ is given as follows,

(21) $$\begin{array}{lll} {\hat{\theta }}^{EBS1} & = & \int_{\varrho }\int {\hat{a}}^{BS}\left(a,b\right)\pi_{1}\left(a,b\right)dbda,\\ & = & \frac{1}{sB(u,v)}\int_{0}^{1}\int_{0}^{s}\left[\omega \left(\frac{r}{T}\right)+\left(1-w\right)\left(\frac{r+a}{b+T}\right)\right]a^{u-1}{\left(1-a\right)}^{v-1}dbda.\\ & = & \frac{\omega r}{T}+\frac{1-w}{s}\left(r+\frac{u}{u+v}\right)\ln\left(\frac{s+T}{T}\right). \end{array}$$

Similarly, the E-Bayesian estimators of θ under BSEL and based on $\pi_{2}\left(a,b\right)$ and $\pi_{3}\left(a,b\right)$ are computed and given, respectively, by

(22) $$\begin{array}{ccc} {\hat{\theta }}^{EBS2} & = & \frac{\omega r}{T}+\frac{2(1-w)}{s}\left(r+\frac{u}{u+v}\right)\left[\frac{s+T}{s}\ln\left(\frac{s+T}{T}\right)-1\right], \end{array}$$

and

(23) $$\begin{array}{ccc} {\hat{\theta }}^{EBS3} & = & \frac{\omega r}{T}+\frac{2(1-w)}{s}\left(r+\frac{u}{u+v}\right)\left[1-\frac{T}{s}\ln\left(\frac{s+T}{T}\right)\right]. \end{array}$$

### 3.2. E-Bayesian Estimation of the Reliability Function

In this subsection, the closed-form of the E-Bayesian estimator for the reliability function of the two-parameter lifetime distribution are discussed. Using Equations (16), (19) and (20), the E-Bayesian estimators of the reliability function under the BSEL function and based on $\pi_{1}\left(a,b\right)$ can be obtained as

(24) $$\begin{array}{lll} {\hat{R}}^{EBS1} & = & \int_{\varrho }\int {\hat{R}}^{BS}\left(t\right)\pi_{1}\left(a,b\right)dbda\\ & = & \frac{1}{sB(u,v)}\int_{0}^{1}\int_{0}^{s}\left[\omega e^{\frac{r}{T}\left(1-e^{t^{\lambda }}\right)}+\left(1-w\right){\left(\frac{b+T}{b+T+T^{*}}\right)}^{r+a}\right]a^{u-1}{\left(1-a\right)}^{v-1}dbda.\\ & = & \omega e^{\frac{r}{T}\left(1-e^{t^{\lambda }}\right)}+\frac{1-w}{sB(u,v)}\int_{0}^{s}{\left(\frac{b+T}{b+T+T^{*}}\right)}^{r}\left\{\int_{0}^{1}e^{a\ln\left(\frac{b+T}{b+T+T^{*}}\right)}a^{u-1}{\left(1-a\right)}^{v-1}da\right\}db.\\ & = & \omega e^{\frac{r}{T}\left(1-e^{t^{\lambda }}\right)}+\frac{1-w}{sB(u,v)}\int_{0}^{s}{\left(\frac{b+T}{b+T+T^{*}}\right)}^{r}F_{1:1}\left(u,u+v;\ln\left(\frac{b+T}{b+T+T^{*}}\right)\right)db, \end{array}$$

where $F_{1:1}\left(\cdot ,\cdot ;\cdot \right)$ is the generalized hypergeometric function [see, Reference [19], (formula 3.383(1))]. Similarly, the E-Bayesian estimators of the reliability function under BSEL and based on $\pi_{2}\left(a,b\right)$ and $\pi_{3}\left(a,b\right)$ are computed and given, respectively, by

(25) $$\begin{array}{ccc} {\hat{R}}^{EBS2} & = & \omega e^{\frac{r}{T}\left(1-e^{t^{\lambda }}\right)}+\frac{2(1-w)}{s^{2}B\left(u,v\right)}\int_{0}^{s}\left(s-b\right){\left(\frac{b+T}{b+T+T^{*}}\right)}^{r}F_{1:1}\left(u,u+v;\ln\left(\frac{b+T}{b+T+T^{*}}\right)\right)db, \end{array}$$

and

(26) $$\begin{array}{ccc} {\hat{R}}^{EBS3} & = & \omega e^{\frac{r}{T}\left(1-e^{t^{\lambda }}\right)}+\frac{2(1-w)}{s^{2}B\left(u,v\right)}\int_{0}^{s}b{\left(\frac{b+T}{b+T+T^{*}}\right)}^{r}F_{1:1}\left(u,u+v;\ln\left(\frac{b+T}{b+T+T^{*}}\right)\right)db. \end{array}$$

The double integrals in Equations (24)–(26) cannot be computed analytically, therefore, it may be derived numerically using mathematical packages such as Maple.

### 3.3. E-Bayesian Estimation of the Hazard Rate Function

In this subsection, the closed forms of the E-Bayesian estimators for the hazard rate function of the two-parameters lifetime distribution are discussed. Using Equations (18)–(20), the E-Bayesian estimator of the hazard rate function under BSEL function and based on $\pi_{1}\left(a,b\right)$ can be obtained as

(27) $$\begin{array}{lll} {\hat{h}}^{EBS1} & = & \int_{\varrho }\int {\hat{h}}^{BS}\left(t\right)\pi_{1}\left(a,b\right)dbda\\ & = & \frac{1}{sB(u,v)}\int_{0}^{1}\int_{0}^{s}\lambda t^{\lambda -1}e^{t^{\lambda }}\left[\omega \left(\frac{r}{T}\right)+\left(1-w\right)\left(\frac{r+a}{b+T}\right)\right]a^{u-1}{\left(1-a\right)}^{v-1}dbda.\\ & = & \lambda t^{\lambda -1}e^{t^{\lambda }}\left[\left(\frac{\omega r}{T}\right)+\frac{\left(1-w\right)}{sB(u,v)}\int_{0}^{1}\int_{0}^{s}\left(\frac{r+a}{b+T}\right)a^{u-1}{\left(1-a\right)}^{v-1}dbda\right].\\ & = & \lambda t^{\lambda -1}e^{t^{\lambda }}\left[\frac{\omega r}{T}+\frac{1-w}{s}\left(r+\frac{u}{u+v}\right)\ln\left(\frac{s+T}{T}\right)\right], \end{array}$$

Similarly, we can obtain the E-Bayesian estimators of the hazard rate function under the BSEL function and based on $\pi_{2}\left(a,b\right)$ and $\pi_{3}\left(a,b\right)$, respectively, as

(28) $$\begin{array}{ccc} {\hat{h}}^{EBS2} & = & \lambda t^{\lambda -1}e^{t^{\lambda }}\left[\frac{\omega r}{T}+\frac{2(1-w)}{s}\left(r+\frac{u}{u+v}\right)\left(\frac{s+T}{s}\ln\left(\frac{s+T}{T}\right)-1\right)\right], \end{array}$$

and

(29) $$\begin{array}{ccc} {\hat{h}}^{EBS3} & = & \lambda t^{\lambda -1}e^{t^{\lambda }}\left[\frac{\omega r}{T}+\frac{2(1-w)}{s}\left(r+\frac{u}{u+v}\right)\left(1-\frac{T}{s}\ln\left(\frac{s+T}{T}\right)\right)\right]. \end{array}$$

## 4. Properties of E-Bayesian Estimation Based on BSEL

In this section, the relationships between ${\hat{\theta }}^{EBSi}$, ${\hat{R}}^{EBSi}$ and ${\hat{h}}^{EBSi}$ (i=1,2,3) will be discussed.

**I**. Relations among ${\hat{\theta }}^{EBSi}$ (i=1,2,3):
  **Proposition** **1.** *Let 0<s<T, u>0, v>0 and ${\hat{\theta }}^{EBSi}$ (i=1,2,3) are given by Equations (21)–(23), then we have the following*
  (i) ${\hat{\theta }}^{EBS2}<{\hat{\theta }}^{EBS1}<{\hat{\theta }}^{EBS3}$;
  (ii) $\lim_{T\to \infty }{\hat{\theta }}^{EBS1}=\lim_{T\to \infty }{\hat{\theta }}^{EBS2}=\lim_{T\to \infty }{\hat{\theta }}^{EBS3}$.
  **Proof.** See Appendix A. □
**II**. Relations among ${\hat{R}}^{EBSi}$ (i=1,2,3):
  **Proposition** **2.** *Let 0<s<T, u>0, v>0 and ${\hat{R}}^{EBSi}$ (i=1,2,3) are given by Equations (24)–(26), then*
  $$\lim_{T\to \infty }{\hat{R}}^{EBS1}=\lim_{T\to \infty }{\hat{R}}^{EBS2}=\lim_{T\to \infty }{\hat{R}}^{EBS3}.$$
  **Proof.** See Appendix A. □
  From Equations (24)–(26), we have
  $$\begin{array}{lll} {\hat{R}}^{EBS3}-{\hat{R}}^{EBS1} & = & {\hat{R}}^{EBS1}-{\hat{R}}^{EBS2}\\ & = & \frac{1-\omega }{s^{2}}\int_{0}^{s}\left(2b-s\right){\left(\frac{b+T}{b+T+T^{*}}\right)}^{r}F_{1:1}\left(u,u+v;\ln\left(\frac{b+T}{b+T+T^{*}}\right)\right)db\\ & > & 0, \end{array}$$
  implying
  $${\hat{R}}^{EBS2}<{\hat{R}}^{EBS1}<{\hat{R}}^{EBS3}.$$
  The integrals in Equations (24)–(26) cannot be computed analytically, therefore, it will be obtained numerically using the mathematical packages Maple.
**III**. Relations among ${\hat{h}}^{EBSi}$ (i=1,2,3):
  **Proposition** **3.** *Let 0<s<T, u>0, v>0 and ${\hat{h}}^{EBSi}$ (i=1,2,3) are given by Equations (27)–(29), then*
  (i) ${\hat{h}}^{EBS3}<{\hat{h}}^{EBS1}<{\hat{h}}^{EBS2}$;
  (ii) $\lim_{T\to \infty }{\hat{h}}^{EBS1}=\lim_{T\to \infty }{\hat{h}}^{EBS2}=\lim_{T\to \infty }{\hat{h}}^{EBS3}$.
  **Proof.** See Appendix A. □

## 5. Monte Carlo Simulation and Comparisons

In this section, a Monte Carlo simulation study is conducted to compare different estimators of the scale parameter, reliability and hazard functions. The simulation study is conducted according to the following steps:

1. Determine the sample size n=20, 50 or 100 and the parameters (θ,λ)=(0.4,0.8) or (0.5, 1.5) and the type-I censoring time τ=0.5 or 1.5;
2. Determine the values (u,v)=(0.5,0.5), s=0.5 or 10 and ω=0.3;
3. The Bayesian and E-Bayesian estimates are calculated by using two types of priors:
  - Prior I: (a,b)=(2.0,1.0);
  - Prior II: (a,b)=(1.5,0.5);
4. For given sample size *n* and censoring time τ, generate $X_{1},\ldots ,X_{n}$ from
  $X_{i}={\left(\log\left(1-\frac{1}{\theta }\log\left(1-U_{i}\right)\right)\right)}^{1/\lambda },\;\;i=1,\ldots ,n$, where $U_{i}$ is uniform (0,1), and consider only $X_{\left(1\right)},...,X_{\left(r\right)}$, where $X_{\left(r\right)}<\tau$;
5. Under the BSEL function, the estimates ${\hat{\theta }}^{BS}$ and ${\hat{\theta }}^{EBSi}$, i=1,2,3 are computed from Equations (15) and (21)–(23), respectively;
6. Under the BSEL function, the estimates ${\hat{R}}^{BS}$ and ${\hat{R}}^{EBSi}$, i=1,2,3 are computed at x=0.5 from Equations (16) and (24)–(26), respectively;
7. Under the BSEL function, the estimates ${\hat{h}}^{BS}$ and ${\hat{h}}^{EBSi}$, i=1,2,3 are computed at x=0.5 from Equations (18) and (27)–(29), respectively.
8. Repeat Steps 4–7 10,000 times. The average estimates (AEs) and the mean squared errors (MSEs) of the 10,000 of estimates under different settings are calculated and summarized.
9. The computational results are displayed in Table 1, Table 2, Table 3 and Table 4.

From Table 1, Table 2, Table 3 and Table 4 we have the following observations:

(1) The differences between AEs and the true value, and the MSEs of the different estimates decrease as *n* increases.
(2) The differences between AEs and the true value, and the MSEs of the different estimates decrease as *s* increases.
(3) The differences between AEs and the true value, and the MSEs of the different estimates in Prior I are less than Prior II.
(4) The E-Bayesian estimates of θ perform better than Bayesian estimates in terms of minimum MSE.
(5) The E-Bayesian estimates of θ have the minimum MSE among all other estimates.
(6) The E-Bayesian estimates of θ based on BSEL loss function with prior distribution $\pi_{1}\left(a,b\right)$ have the minimum MSE comparing with all other estimates.

Combining all the above results, we recommend using the E-Bayesian procedure to estimate the scale parameter, reliability and hazard rate functions of Chen distribution based on the type-I censoring scheme using prior distribution $\pi_{1}\left(a,b\right)$, which performs better than other estimates in terms of minimum MSE.

## 6. Real Data Analysis

In this section, we analyze a real data set, which was originally reported in Reference [20]. The dataset contains the graft survival times in months of 148 renal transplant patients. The complete dataset is presented in Table 5, which consists of 148 observations. Reference [21] reported that the Chen distribution provides a good fit to this dataset, which has a bathtub failure rate shape. The MLEs of the unknown parameters of the Chen distribution are obtained as $\hat{\theta }=0.0429$ and $\hat{\lambda }=0.3863$. From the complete dataset in Table 5, we generate two type-I censored samples by choosing τ to be 10 and 20. The number of observations in these samples are 52 and 93, respectively. We consider that the shape parameter is known in all the cases and equal to its MLE, i.e., $\hat{\lambda }=0.3863$. Different estimates of the parameter θ, reliability and hazard rate functions are obtained and presented in Table 6. The reliability and hazard rate functions are estimated at 15 months. The Bayes estimates are obtained under the case of noninformative priors when there is no prior information about the unknown parameters. The hyper-parameters in this case are selected to be 0.1. In addition, the approximated MSEs are obtained by considering the estimates based on the complete dataset as the true values of the parameters. From the results in Table 6, it is noted that the approximated MSEs decrease as the censoring time τ increases. We can also observe that the E-Bayesian estimates perform better than the MLEs and Bayes estimates in terms of the approximated MSEs.

## 7. Concluding Remarks

In this paper, we have studied the E-Bayesian estimation of the scale parameter, reliability and hazard rate functions of the two-parameter bathtub-shape distribution proposed by Chen (2000) based on the type-I censored sample. The E-Bayesian estimators are obtained based on the balanced squared error loss function and by considering three prior distributions of the hyper-parameters. Some properties of the E-Bayesian estimators are discussed. A simulation study is conducted to compare the efficiencies of the Bayesian estimators with the proposed E-Bayesian estimators based on mean squared errors. The simulation results showed that the E-Bayesian estimators perform better than the Bayesian estimators based on minimum mean squared errors. A real data set is analyzed to illustrate how to obtain the proposed estimators in practice. The results of this analysis agree with the simulation results. As a future work, we suggest obtaining the E-Bayesian estimators of the Chen distribution when both the parameters in the model are unknown.

## Figures and Tables

**Table 1:** Simulated average estimates (AEs) (first row) and mean squared errors (MSEs) (second row) under different settings with θ=0.4, λ=0.8.

*n*	Par	MLE	Prior I				Prior II			
			**BS**	**EBS1**	**EBS2**	**EBS3**	**BS**	**EBS1**	**EBS2**	**EBS3**
τ=1.5, s=0.5										
20	θ	0.4116	0.4428	0.4196	0.4203	0.4188	0.4401	0.4222	0.4229	0.4214
		0.0133	0.0154	0.0136	0.0137	0.0137	0.0141	0.0126	0.0127	0.0125
	*R*	0.7294	0.7121	0.7268	0.7264	0.7272	0.7133	0.7251	0.7247	0.7255
		0.0040	0.0043	0.0039	0.0039	0.0039	0.0040	0.0036	0.0036	0.0036
	*h*	0.6718	0.7226	0.6848	0.6860	0.6836	0.7183	0.6890	0.6902	0.6878
		0.0353	0.0410	0.0363	0.0366	0.0360	0.0376	0.0336	0.0339	0.0333
50	θ	0.4058	0.4185	0.4090	0.4093	0.4087	0.4191	0.4118	0.4121	0.4116
		0.0053	0.0056	0.0053	0.0053	0.0053	0.0056	0.0053	0.0053	0.0053
	*R*	0.7310	0.7238	0.7299	0.7298	0.7301	0.7235	0.7283	0.7282	0.7285
		0.0016	0.0017	0.0016	0.0016	0.0016	0.0017	0.0016	0.0016	0.0016
	*h*	0.6623	0.6830	0.6675	0.6680	0.6671	0.6839	0.6721	0.6726	0.6717
		0.0140	0.0150	0.0142	0.0142	0.0141	0.0148	0.0141	0.0141	0.0140
100	θ	0.4018	0.4082	0.4034	0.4036	0.4033	0.4083	0.4047	0.4048	0.4045
		0.0022	0.0023	0.0023	0.0023	0.0023	0.0024	0.0023	0.0023	0.0023
	*R*	0.7326	0.7290	0.7321	0.7320	0.7321	0.7290	0.7314	0.7313	0.7314
		0.0007	0.0007	0.0007	0.0007	0.0007	0.0007	0.0007	0.0007	0.0007
	*h*	0.6558	0.6662	0.6584	0.6587	0.6582	0.6663	0.6604	0.6607	0.6602
		0.0060	0.0062	0.0060	0.0060	0.0060	0.0063	0.0061	0.0061	0.0061
τ=1.5, s=10										
20	θ	0.4108	0.4419	0.3834	0.3948	0.3721	0.4421	0.3394	0.3623	0.3165
		0.0137	0.0157	0.0108	0.0115	0.0104	0.0161	0.0111	0.0103	0.0131
	*R*	0.7300	0.7127	0.7467	0.7403	0.7530	0.7126	0.7725	0.7590	0.7859
		0.0041	0.0044	0.0035	0.0036	0.0035	0.0045	0.0040	0.0035	0.0049
	*h*	0.6704	0.7211	0.6258	0.6444	0.6072	0.7215	0.5539	0.5912	0.5166
		0.0364	0.0419	0.0287	0.0306	0.0277	0.0430	0.0295	0.0273	0.0348
50	θ	0.4040	0.4167	0.3924	0.3974	0.3874	0.4120	0.3651	0.3773	0.3529
		0.0046	0.0050	0.0042	0.0043	0.0042	0.0047	0.0046	0.0042	0.0053
	*R*	0.7319	0.7248	0.7391	0.7363	0.7419	0.7274	0.7549	0.7478	0.7620
		0.0015	0.0015	0.0014	0.0014	0.0014	0.0015	0.0016	0.0014	0.0019
	*h*	0.6593	0.6800	0.6404	0.6486	0.6323	0.6724	0.5958	0.6157	0.5760
		0.0123	0.0132	0.0112	0.0115	0.0111	0.0126	0.0122	0.0112	0.0141
100	θ	0.4009	0.4073	0.3951	0.3977	0.3925	0.4068	0.3815	0.3885	0.3746
		0.0023	0.0024	0.0022	0.0022	0.0022	0.0023	0.0022	0.0021	0.0024
	*R*	0.7331	0.7295	0.7368	0.7353	0.7383	0.7298	0.7446	0.7406	0.7486
		0.0007	0.0008	0.0007	0.0007	0.0007	0.0007	0.0008	0.0007	0.0008
	*h*	0.6544	0.6648	0.6448	0.6490	0.6406	0.6639	0.6227	0.6340	0.6113
		0.0061	0.0063	0.0059	0.0059	0.0059	0.0061	0.0059	0.0057	0.0065

**Table 2:** Simulated AEs (first row) and MSEs (second row) under different settings with θ=0.5, λ=1.5.

*n*	Par	MLE	Prior I				Prior II			
			**BS**	**EBS1**	**EBS2**	**EBS3**	**BS**	**EBS1**	**EBS2**	**EBS3**
τ=1.5, s=0.5										
20	θ	0.5165	0.5440	0.5235	0.5244	0.5226	0.5493	0.5326	0.5335	0.5317
		0.0163	0.0186	0.0167	0.0169	0.0166	0.0184	0.0164	0.0166	0.0163
	*R*	0.8044	0.7951	0.8028	0.8025	0.8031	0.7933	0.7997	0.7994	0.8000
		0.0018	0.0020	0.0018	0.0018	0.0018	0.0020	0.0018	0.0018	0.0017
	*h*	0.7802	0.8217	0.7908	0.7921	0.7895	0.8297	0.8045	0.8059	0.8032
		0.0371	0.0424	0.0382	0.0385	0.0378	0.0420	0.0375	0.0378	0.0371
50	θ	0.5103	0.5215	0.5131	0.5135	0.5128	0.5201	0.5135	0.5138	0.5131
		0.0061	0.0066	0.0062	0.0063	0.0062	0.0066	0.0063	0.0063	0.0063
	*R*	0.8058	0.8020	0.8052	0.8050	0.8053	0.8025	0.8050	0.8049	0.8052
		0.0007	0.0007	0.0007	0.0007	0.0007	0.0008	0.0007	0.0007	0.0007
	*h*	0.7708	0.7877	0.7751	0.7756	0.7746	0.7856	0.7756	0.7761	0.7751
		0.0140	0.0151	0.0142	0.0143	0.0142	0.0152	0.0144	0.0145	0.0144
100	θ	0.5040	0.5096	0.5054	0.5056	0.5052	0.5077	0.5044	0.5046	0.5042
		0.0026	0.0027	0.0026	0.0026	0.0026	0.0029	0.0029	0.0029	0.0029
	*R*	0.8077	0.8058	0.8074	0.8073	0.8075	0.8065	0.8078	0.8077	0.8078
		0.0003	0.0003	0.0003	0.0003	0.0003	0.0003	0.0003	0.0003	0.0003
	*h*	0.7613	0.7698	0.7634	0.7637	0.7632	0.7669	0.7619	0.7622	0.7617
		0.0060	0.0062	0.0060	0.0060	0.0060	0.0067	0.0065	0.0065	0.0065
τ=1.5, s=10										
20	θ	0.4140	0.4453	0.3863	0.3979	0.3748	0.4427	0.3887	0.4004	0.3771
		0.0139	0.0161	0.0108	0.0116	0.0103	0.0150	0.0099	0.0107	0.0095
	*R*	0.7281	0.7108	0.7451	0.7387	0.7515	0.7120	0.7435	0.7370	0.7499
		0.0041	0.0045	0.0034	0.0035	0.0034	0.0042	0.0032	0.0033	0.0031
	*h*	0.6757	0.7268	0.6305	0.6493	0.6117	0.7226	0.6345	0.6534	0.6155
		0.0370	0.0430	0.0287	0.0308	0.0275	0.0399	0.0264	0.0284	0.0253
50	θ	0.4024	0.4151	0.3910	0.3960	0.3860	0.4119	0.3901	0.3951	0.3852
		0.0045	0.0048	0.0042	0.0042	0.0041	0.0049	0.0043	0.0044	0.0042
	*R*	0.7328	0.7256	0.7399	0.7371	0.7427	0.7274	0.7404	0.7376	0.7432
		0.0014	0.0015	0.0014	0.0014	0.0014	0.0015	0.0014	0.0014	0.0014
	*h*	0.6568	0.6775	0.6381	0.6462	0.6300	0.6723	0.6367	0.6448	0.6286
		0.0121	0.0128	0.0111	0.0113	0.0110	0.0130	0.0114	0.0116	0.0113
100	θ	0.4035	0.4099	0.3976	0.4002	0.3950	0.4087	0.3976	0.4002	0.3950
		0.0024	0.0025	0.0022	0.0023	0.0022	0.0025	0.0022	0.0023	0.0022
	*R*	0.7317	0.7281	0.7354	0.7339	0.7368	0.7287	0.7354	0.7339	0.7368
		0.0008	0.0008	0.0007	0.0007	0.0007	0.0008	0.0007	0.0007	0.0007
	*h*	0.6586	0.6690	0.6489	0.6531	0.6447	0.6671	0.6489	0.6531	0.6447
		0.0063	0.0066	0.0060	0.0061	0.0059	0.0065	0.0060	0.0061	0.0059

**Table 3:** Simulated AEs (first row) and MSEs (second row) under different settings with θ=0.4, λ=0.8.

*n*	Par	MLE	Prior I				Prior II			
			**BS**	**EBS1**	**EBS2**	**EBS3**	**BS**	**EBS1**	**EBS2**	**EBS3**
τ=2.5, s=0.5										
20	θ	0.4181	0.4417	0.4241	0.4247	0.4235	0.4384	0.4247	0.4253	0.4242
		0.0102	0.0122	0.0106	0.0107	0.0105	0.0125	0.0111	0.0112	0.0111
	*R*	0.7250	0.7120	0.7231	0.7228	0.7234	0.7140	0.7229	0.7226	0.7232
		0.0030	0.0034	0.0030	0.0030	0.0030	0.0035	0.0031	0.0032	0.0031
	*h*	0.6824	0.7209	0.6922	0.6931	0.6912	0.7155	0.6932	0.6941	0.6923
		0.0271	0.0324	0.0282	0.0284	0.0280	0.0332	0.0297	0.0299	0.0294
50	θ	0.4082	0.4178	0.4106	0.4109	0.4104	0.4137	0.4083	0.4086	0.4081
		0.0037	0.0040	0.0037	0.0038	0.0037	0.0039	0.0037	0.0037	0.0037
	*R*	0.7293	0.7239	0.7285	0.7284	0.7286	0.7262	0.7298	0.7296	0.7299
		0.0011	0.0012	0.0011	0.0012	0.0011	0.0012	0.0011	0.0011	0.0011
	*h*	0.6663	0.6818	0.6702	0.6705	0.6698	0.6752	0.6664	0.6668	0.6661
		0.0098	0.0107	0.0100	0.0100	0.0099	0.0104	0.0099	0.0099	0.0098
100	θ	0.4030	0.4077	0.4042	0.4043	0.4041	0.4065	0.4038	0.4039	0.4037
		0.0018	0.0019	0.0018	0.0018	0.0018	0.0018	0.0018	0.0018	0.0018
	*R*	0.7319	0.7292	0.7314	0.7314	0.7315	0.7299	0.7317	0.7316	0.7317
		0.0006	0.0006	0.0006	0.0006	0.0006	0.0006	0.0006	0.0006	0.0006
	*h*	0.6577	0.6655	0.6596	0.6598	0.6595	0.6634	0.6590	0.6591	0.6588
		0.0047	0.0049	0.0048	0.0048	0.0048	0.0048	0.0047	0.0047	0.0047
τ=2.5, s=10										
20	θ	0.4205	0.4442	0.3979	0.4073	0.3885	0.4376	0.3958	0.4051	0.3865
		0.0111	0.0132	0.0083	0.0091	0.0078	0.0120	0.0078	0.0085	0.0073
	*R*	0.7238	0.7108	0.7376	0.7324	0.7428	0.7143	0.7386	0.7335	0.7438
		0.0032	0.0037	0.0026	0.0027	0.0025	0.0034	0.0024	0.0026	0.0023
	*h*	0.6863	0.7250	0.6494	0.6647	0.6340	0.7143	0.6459	0.6611	0.6308
		0.0295	0.0352	0.0222	0.0243	0.0207	0.0319	0.0208	0.0227	0.0195
50	θ	0.4051	0.4146	0.3962	0.4001	0.3924	0.4135	0.3968	0.4007	0.3930
		0.0035	0.0038	0.0032	0.0033	0.0031	0.0039	0.0033	0.0034	0.0032
	*R*	0.7310	0.7257	0.7365	0.7344	0.7387	0.7263	0.7362	0.7340	0.7384
		0.0011	0.0012	0.0010	0.0010	0.0010	0.0012	0.0011	0.0011	0.0010
	*h*	0.6611	0.6766	0.6467	0.6530	0.6404	0.6749	0.6477	0.6540	0.6414
		0.0094	0.0101	0.0085	0.0087	0.0083	0.0104	0.0088	0.0091	0.0086
100	θ	0.4022	0.4070	0.3978	0.3997	0.3958	0.4074	0.3991	0.4010	0.3971
		0.0017	0.0018	0.0016	0.0016	0.0016	0.0020	0.0018	0.0018	0.0018
	*R*	0.7323	0.7296	0.7350	0.7339	0.7361	0.7294	0.7344	0.7332	0.7355
		0.0005	0.0006	0.0005	0.0005	0.0005	0.0006	0.0006	0.0006	0.0006
	*h*	0.6564	0.6642	0.6492	0.6524	0.6460	0.6649	0.6513	0.6545	0.6481
		0.0045	0.0047	0.0043	0.0044	0.0042	0.0052	0.0048	0.0049	0.0047

**Table 4:** Simulated AEs (first row) and MSEs (second row) under different settings with θ=0.5, λ=1.5.

*n*	Par	MLE	Prior I				Prior II			
			**BS**	**EBS1**	**EBS2**	**EBS3**	**BS**	**EBS1**	**EBS2**	**EBS3**
τ=2.5, s=0.5										
20	α	0.5286	0.5545	0.5352	0.5361	0.5343	0.5548	0.5390	0.5399	0.5381
		0.0176	0.0206	0.0182	0.0184	0.0180	0.0207	0.0184	0.0186	0.0182
	*R*	0.8003	0.7917	0.7989	0.7986	0.7991	0.7916	0.7976	0.7973	0.7979
		0.0019	0.0022	0.0019	0.0019	0.0019	0.0022	0.0019	0.0019	0.0019
	*h*	0.7984	0.8376	0.8084	0.8097	0.8071	0.8380	0.8142	0.8155	0.8129
		0.0401	0.0470	0.0416	0.0420	0.0412	0.0471	0.0420	0.0424	0.0415
50	α	0.5054	0.5158	0.5080	0.5083	0.5077	0.5190	0.5129	0.5132	0.5125
		0.0048	0.0051	0.0049	0.0049	0.0048	0.0060	0.0057	0.0057	0.0057
	*R*	0.8074	0.8039	0.8068	0.8067	0.8069	0.8028	0.8052	0.8051	0.8053
		0.0006	0.0006	0.0006	00.0006	0.0006	0.0007	0.0006	0.0006	0.0006
	*h*	0.7634	0.7791	0.7674	0.7678	0.7669	0.7840	0.7747	0.7751	0.7742
		0.0109	0.0117	0.0111	0.0111	0.0110	0.0137	0.0130	0.0131	0.0130
100	α	0.5050	0.5103	0.5063	0.5065	0.5062	0.5087	0.5057	0.5058	0.5055
		0.0025	0.0026	0.0025	0.0025	0.0025	0.0025	0.0024	0.0024	0.0024
	*R*	0.8074	0.8056	0.8071	0.8070	0.8071	0.8061	0.8073	0.8072	0.8073
		0.0003	0.0003	0.0003	0.0003	0.0003	0.0003	0.0003	0.0003	0.0003
	*h*	0.7628	0.7708	0.7648	0.7651	0.7646	0.7684	0.7638	0.7640	0.7636
		0.0057	0.0059	0.0057	0.0057	0.0057	0.0057	0.0056	0.0056	0.0056
τ=2.5, s=10										
20	α	0.5391	0.5654	0.5024	0.5165	0.4884	0.5452	0.4887	0.5020	0.4754
		0.0179	0.0215	0.0119	0.0136	0.0108	0.0178	0.0111	0.0121	0.0104
	*R*	0.7968	0.7880	0.8097	0.8049	0.8144	0.7947	0.8143	0.8098	0.8188
		0.0019	0.0023	0.0013	0.0015	0.0012	0.0019	0.0013	0.0014	0.0012
	*h*	0.8143	0.8540	0.7589	0.7802	0.7377	0.8235	0.7382	0.7583	0.7181
		0.0408	0.0490	0.0272	0.0309	0.0246	0.0406	0.0252	0.0277	0.0237
50	α	0.5120	0.5224	0.4979	0.5036	0.4921	0.5185	0.4957	0.5014	0.4901
		0.0062	0.0067	0.0053	0.0056	0.0051	0.0059	0.0048	0.0050	0.0046
	*R*	0.8053	0.8017	0.8103	0.8084	0.8122	0.8030	0.8110	0.8091	0.8129
		0.0007	0.0008	0.0006	0.0006	0.0006	0.0007	0.0006	0.0006	0.0005
	*h*	0.7733	0.7892	0.7520	0.7606	0.7434	0.7831	0.7488	0.7573	0.7402
		0.0140	0.0152	0.0121	0.0127	0.0117	0.0134	0.0109	0.0114	0.0106
100	α	0.5032	0.5085	0.4963	0.4992	0.4935	0.5116	0.5001	0.5030	0.4972
		0.0024	0.0025	0.0023	0.0023	0.0023	0.0026	0.0023	0.0024	0.0023
	*R*	0.8080	0.8062	0.8105	0.8095	0.8115	0.8051	0.8092	0.8082	0.8102
		0.0003	0.0003	0.0003	0.0003	0.0003	0.0003	0.0003	0.0003	0.0003
	*h*	0.7601	0.7680	0.7497	0.7540	0.7454	0.7727	0.7554	0.7598	0.7510
		0.0055	0.0057	0.0052	0.0053	0.0051	0.0060	0.0053	0.0054	0.0052

**Table 5:** The graft survival times in months of renal transplant patients.

0.0350	0.0680	0.1000	0.1010	0.1670	0.1680	0.1970	0.2130	0.2330	0.2340
0.5080	0.5080	0.5330	0.6330	0.7670	0.7680	0.7700	1.0660	1.2670	1.3000
1.6000	1.6390	1.8030	1.8670	2.1800	2.6670	2.9670	3.3280	3.3930	3.7000
3.8030	4.3110	4.8670	5.1800	6.2330	6.3670	6.6000	6.6000	7.1800	7.6670
7.7330	7.8000	7.9330	7.9670	8.0160	8.3000	8.4100	8.6070	8.6670	8.8000
9.1000	9.2330	10.541	10.607	10.633	10.667	10.869	11.067	11.180	11.443
12.213	12.508	12.533	13.467	13.800	14.267	14.475	14.500	15.213	15.333
15.525	15.533	15.541	15.934	16.200	16.300	16.344	16.600	16.700	16.933
17.033	17.067	17.475	17.667	17.700	17.967	18.115	18.115	18.933	18.934
19.508	19.574	19.733	20.148	20.180	20.900	21.167	21.233	21.600	22.100
22.148	22.180	22.180	22.267	22.300	22.500	22.533	22.867	23.738	24.082
24.180	24.705	25.213	25.705	29.705	30.443	31.667	31.934	32.180	32.367
32.672	32.705	33.148	33.567	33.770	33.869	34.836	34.869	34.934	35.738
36.180	36.213	39.410	39.433	39.672	40.001	41.733	41.734	42.311	42.869
43.180	43.279	43.902	44.267	44.475	44.900	45.148	46.451		

**Table 6:** Different estimates and the corresponding approximated MSEs of real data set.

τ	Par	MLEs	BS	EBS1	EBS2	EBS3
10	θ	0.04267	0.04272	0.04311	0.04315	0.04307
	Approx. MSE	$5.48610500\times 10^{-8}$	$3.21284\times 10^{-8}$	$4.42096\times 10^{-8}$	$6.32734\times 10^{-8}$	$2.85541\times 10^{-8}$
	*R*	0.50036	0.49992	0.49838	0.49805	0.49871
	Approx. MSE	$3.84119\times 10^{-6}$	$2.29110\times 10^{-6}$	$6.53381\times 10^{-10}$	$1.25679\times 10^{-7}$	$9.20456\times 10^{-8}$
	*h*	0.05389	0.05396	0.05445	0.05450	0.05440
	Approx. MSE	$9.67848\times 10^{-8}$	$5.83999\times 10^{-8}$	$6.26477\times 10^{-8}$	$9.14667\times 10^{-8}$	$3.92657\times 10^{-8}$
20	θ	0.04279	0.04282	0.04304	0.04306	0.04301
	Approx. MSE	$1.31291\times 10^{-8}$	$7.01528\times 10^{-8}$	$6.37777\times 10^{-8}$	$4.39350\times 10^{-8}$	$1.24892\times 10^{-8}$
	*R*	0.49939	0.49914	0.49828	0.49809	0.49846
	Approx. MSE	$9.78738\times 10^{-7}$	$5.46935\times 10^{-7}$	$1.51927\times 10^{-8}$	$9.51861\times 10^{-8}$	$3.84462\times 10^{-9}$
	*h*	0.05404	0.05408	0.05435	0.05438	0.05433
	Approx. MSE	$2.55972\times 10^{-8}$	$1.46551\times 10^{-8}$	$2.40434\times 10^{-8}$	$3.39441\times 10^{-8}$	$1.58456\times 10^{-8}$

## Appendix A

**Proof** **of** **Proposition** **1.**

(i) From Equations (21)–(23), we have
  (A1) $$\begin{array}{lll} {\hat{\theta }}^{EBS2}-{\hat{\theta }}^{EBS1} & = & {\hat{\theta }}^{EBS1}-{\hat{\theta }}^{EBS3}\\ & = & \frac{1-\omega }{s}\left(r+\frac{u}{u+v}\right)\left[\frac{s+2T}{s}\ln\left(\frac{T+s}{T}\right)-2\right] \end{array}$$
  For −1<x<1, we have: $\ln\left(1+x\right)=x-\frac{x^{2}}{2}+\frac{x^{3}}{3}-\frac{x^{4}}{4}+\ldots =\sum_{k=1}^{\infty }{\left(-1\right)}^{k-1}\frac{x^{k}}{k}.$ Let $x=\frac{s}{T}$, when 0<s<T, $0<\frac{s}{T}<1$, we get:
  (A2) $$\begin{array}{ll} & \left[\frac{s+2T}{s}\ln\left(\frac{T+s}{T}\right)-2\right]\\ = & \frac{s+2T}{s}\left[\left(\frac{s}{T}\right)-\frac{1}{2}{\left(\frac{s}{T}\right)}^{2}+\frac{1}{3}{\left(\frac{s}{T}\right)}^{3}-\frac{1}{4}{\left(\frac{s}{T}\right)}^{4}+\frac{1}{5}{\left(\frac{s}{T}\right)}^{5}-\ldots \right]-2\\ = & \left[\left(\frac{s}{T}\right)-\frac{1}{2}{\left(\frac{s}{T}\right)}^{2}+\frac{1}{3}{\left(\frac{s}{T}\right)}^{3}-\frac{1}{4}{\left(\frac{s}{T}\right)}^{4}+\frac{1}{5}{\left(\frac{s}{T}\right)}^{5}-\ldots \right]-2\\ & +\left(2-\left(\frac{s}{T}\right)+\frac{2}{3}{\left(\frac{s}{T}\right)}^{2}-\frac{2}{4}{\left(\frac{s}{T}\right)}^{3}+\frac{2}{5}{\left(\frac{s}{T}\right)}^{4}-\ldots \right)\\ = & \left(\frac{s^{2}}{6T^{2}}-\frac{s^{3}}{6T^{3}}\right)+\left(\frac{3s^{4}}{6T^{4}}-\frac{2s^{5}}{15T^{5}}\right)+\ldots \\ = & \frac{s^{2}}{6T^{2}}\left(1-\frac{s}{T}\right)+\frac{s^{4}}{60T^{4}}\left(9-\frac{8s}{T}\right)+\ldots \\ > & 0. \end{array}$$
  According to Equations (A1) and (A2), we have
  $${\hat{\theta }}^{EBS2}-{\hat{\theta }}^{EBS1}={\hat{\theta }}^{EBS1}-{\hat{\theta }}^{EBS3}>0,$$
  that is
  $${\hat{\theta }}^{EBS3}<{\hat{\theta }}^{EBS1}<{\hat{\theta }}^{EBS2}.$$
(ii) From Equations (A1) and (A2), we get
  $$\begin{array}{lll} \lim_{T\to \infty }\left({\hat{\theta }}^{EBS2}-{\hat{\theta }}^{EBS1}\right) & = & \lim_{T\to \infty }\left({\hat{\theta }}^{EBS1}-{\hat{\theta }}^{EBS3}\right)\\ & = & \frac{1-\omega }{s}\left(r+\frac{u}{u+v}\right)\lim_{T\to \infty }\left\{\frac{s^{2}}{6T^{2}}\left(1-\frac{s}{T}\right)+\frac{s^{4}}{60T^{4}}\left(9-\frac{8s}{T}\right)+\ldots \right\}\\ & = & 0. \end{array}$$
  That is, $\lim_{T\to \infty }{\hat{\theta }}^{EBS1}=\lim_{T\to \infty }{\hat{\theta }}^{EBS2}=\lim_{T\to \infty }{\hat{\theta }}^{EBS3}$.
  Thus, the proof is complete. □

**Proof** **of** **Proposition** **2.** From Equations (24)–(26), we get

$$\begin{array}{lll} \lim_{T\to \infty }\left({\hat{R}}^{EBS3}-{\hat{R}}^{EBS1}\right) & = & \lim_{T\to \infty }\left({\hat{R}}^{EBS1}-{\hat{R}}^{EBS2}\right)=\lim_{T\to \infty }\{\frac{1-\omega }{s^{2}}\int_{0}^{s}\left(2b-s\right){\left(\frac{b+T}{b+T+T^{*}}\right)}^{r}\\ & & \times F_{1:1}\left(u,u+v;\ln\left(\frac{b+T}{b+T+T^{*}}\right)\right)db\}=0. \end{array}$$

That is, $\lim_{T\to \infty }{\hat{\theta }}^{EBS1}=\lim_{T\to \infty }{\hat{\theta }}^{EBS2}=\lim_{T\to \infty }{\hat{\theta }}^{EBS3}$. Thus, the proof is complete. □

**Proof** **of** **Proposition** **3.**

(i) From Equations (27)–(29), we have
  (A3) $$\begin{array}{lll} {\hat{h}}^{EBS2}-{\hat{h}}^{EBS1} & = & {\hat{h}}^{EBS1}-{\hat{h}}^{EBS3}\\ & = & \lambda t^{\lambda -1}e^{t^{\lambda }}\left(\frac{1-\omega }{s}\right)\left(r+\frac{u}{u+v}\right)\left[\frac{s+2T}{s}\ln\left(\frac{T+s}{T}\right)-2\right] \end{array}$$
  According to Equations (A2) and (A3), we have
  $${\hat{h}}^{EBS2}-{\hat{h}}^{EBS1}={\hat{h}}^{EBS1}-{\hat{h}}^{EBS3}>0,$$
  that is
  $${\hat{h}}^{EBS3}<{\hat{h}}^{EBS1}<{\hat{h}}^{EBS2}.$$
(ii) From Equations (A2) and (A3), we get
  $$\begin{array}{lll} \lim_{T\to \infty }\left({\hat{h}}^{EBS2}-{\hat{h}}^{EBS1}\right) & = & \lim_{T\to \infty }\left({\hat{h}}^{EBS1}-{\hat{h}}^{EBS3}\right)=\lambda t^{\lambda -1}e^{t^{\lambda }}\left(\frac{1-\omega }{s}\right)\left(r+\frac{u}{u+v}\right)\\ & & \times \lim_{T\to \infty }\left\{\frac{s^{2}}{6T^{2}}\left(1-\frac{s}{T}\right)+\frac{s^{4}}{60T^{4}}\left(9-\frac{8s}{T}\right)+\ldots \right\}=0. \end{array}$$
  That is, $\lim_{T\to \infty }{\hat{\theta }}^{EBS1}=\lim_{T\to \infty }{\hat{\theta }}^{EBS2}=\lim_{T\to \infty }{\hat{\theta }}^{EBS3}$.
  Thus, the proof is complete. □
